# Traditional and Current Food Use of Wild Plants Listed in the Russian Pharmacopoeia

**DOI:** 10.3389/fphar.2017.00841

**Published:** 2017-11-21

**Authors:** Alexander N. Shikov, Andrey N. Tsitsilin, Olga N. Pozharitskaya, Valery G. Makarov, Michael Heinrich

**Affiliations:** ^1^Saint-Petersburg Institute of Pharmacy, Kuzmolovsky, Russia; ^2^All Russian Research Institute Medicinal and Aromatic Plants (VILAR), Moscow, Russia; ^3^Research Cluster Biodiversity and Medicines, Centre for Pharmacognosy and Phytotherapy, UCL School of Pharmacy, University of London, London, United Kingdom

**Keywords:** medicinal plants, traditional use, functional food, health benefits, Russian Federation

## Abstract

Historically Russia can be regarded as a “herbophilious” society. For centuries the multinational population of Russia has used plants in daily diet and for self-medication. The specificity of dietary uptake of medicinal plants (especially those in the unique and highly developed Russian herbal medical tradition) has remained mostly unknown in other regions. Based on 11th edition of the State Pharmacopoeia of the USSR, we selected 70 wild plant species which have been used in food by local Russian populations. Empirical searches were conducted via the Russian-wide applied online database E-library.ru, library catalogs of public libraries in St-Petersburg, the databases Scopus, Web of Science, PubMed, and search engine Google Scholar. The large majority of species included in Russian Pharmacopoeia are used as food by local population, however, aerial parts are more widely used for food. In this review, we summarize data on medicinal species published in Russia and other countries that are included in the Russian Pharmacopoeia and have being used in food for a long time. Consequently, the Russian Pharmacopoeia is an important source of information on plant species used traditionally at the interface of food and medicine. At the same time, there are the so-called “functional foods”, which denotes foods that not only serves to provide nutrition but also can be a source for prevention and cure of various diseases. This review highlights the potential of wild species of Russia monographed in its pharmacopeia for further developing new functional foods and—through the lens of their incorporation into the pharmacopeia—showcases the species' importance in Russia.

## Introduction

Until today over 7000 species have been recorded to be edible (Lim, 2012) and for many of these specific health benefits have been claimed, with, however, generally only limited pharmacological evidence for such claims. Only a small share of these is used on a large, extensive scale. Wild and cultivated food plants may have medicinal, nutraceutical, pharmaceutical, and health benefits. Plants are excellent sources of active phytochemicals with importance in the prevention of different diseases. Herbal medicinal products are going recognition for the treatment and prophylaxis of different diseases. Direct nutritional benefits of such plants are often known only to a limited extend. Herbal infusions will add small quantities of essential micronutrients and vitamins to the daily intake. For sick or malnourished individuals this may contribute to restoring the balance between nutrients and thereby improve or sustain health (Lim, 2012).

Blurring of the food and medicine interface is a common theme across multiple contexts and cultures (Heinrich, 2016). The association between food and disease is widely recognized as the bedrock of preventive nutrition. Throughout the ages, and at least since the proclamation “let food be your medicine, let medicine be your food” which has been attributed to Hippocrates' (ca. 480–377 BC), physicians recognized the impact of food on human health. The question of the continuum between food and medicine is of great interest to (ethno-)pharmacologists (Etkin and Ross, 1982; Leonti, 2012; Valussi and Scire, 2012; Jennings et al., 2014; Alarcón et al., 2015).

In many countries medicinal plants are widely used as dietary supplements, in daily foods and as functional foods, with the aim of promoting health. In Eastern cultures food and medicine are seen to come from the same source. Their use is based on the same fundamental theories, and they are equally important in maintaining and improving health and for preventing and curing diseases, as well as facilitating rehabilitation. In China, Japan, Korea, and Southeast Asia, medicinal plants are widely used both daily foods (cereals, vegetables, and fruits) and functional foods, for replacement and medical purposes (Shi et al., 2011). The last few decades has seen the introduction to Europe of a wide range of species used in other continents, including from South Africa e.g. rooibos (*Aspalathus linearis* (Burm.f.) R.Dahlgren) and to a lesser extent honey bush tea (*Cyclopia* sp.) or *Hoodia gordonii* (Masson) Sweet ex Decne. to regulate the appetite. Also some exotic fruits, such as acerola (*Malpighia glabra* L.) from South America, rich in vitamin C are now marketed as food supplements (Franz et al., 2011).

In the early history of Western biomedicine, diet figured prominently in the prevention of diseases and therapeutics. The history of biomedicine customarily is traced to Greece and the Hyppocratic Corpus, some 60 medical texts dating to the fifth to fourth centuries BCE. Diet regulation was the most prominent element in all the Hippocratic texts (Etkin, 2006). Due to its location between East and West Russian tradition of medicine and diet has accumulated and adopted approaches that originated in Europe and Asia. The Greek influence on Russian medicine is of particular relevance in this context (Shikov et al., 2014a).

Russia in the past and present can be regarded as a “herbophilious” society. The term “herbophilia” was used by Łuczaj (2008) for such cultures, in which medicinal and food plant species are often used and highly prized. Approximately 58% of the Russian population was reported to use phytopharmaceuticals as a form of treatment (Shikov et al., 2011) including wild plants. Plants are an integral part of Russian everyday life. A large number of species of green vegetables are used in agricultural communities, particularly those in which food shortages are frequent. Due to the harsh climatic conditions coupled with poor crop harvests Russian peasants were educated on how to survive by relying on wild plants (Kovaleva, 1972).

Wild food plants became highest priority in the USSR during the Second World War most notably during the siege of Leningrad (1941–1944). In 1942 a special manual about most important wild food plants of the Leningrad region was published and distributed to the army and civilians. The scientists from V.L. Komarov Botanical Institute described thirty seven common herbs and two edible lichens, their nutritional and functional properties, specificity of harvesting, and food processing (Tikhomirov, 1942).

As a result of socio-economic changes after the Second World War, the active role of wild plants gradually diminished in regular diet. However, some species are still gathered and made into food products by small companies. Wild berry harvesting is very popular especial among of rural populations. The practice of preparing jam, compotes, and home made liqueurs from wild berries has been passed down by housewives from generation to generation (Grigorieva, 2008).

A wide choice of herbal medicines or botanicals has provided increasing opportunities for both the development and marketing of herbal food products, dietary supplements, and functional foods.

Wild species were selected for food application by humans not only because of their pleasant taste and aroma, but some pharmacological effects were considered as well (Gammerman and Grom, 1976). Herbal medicine products have become popular over the past decade and they are widely used for the treatment and prevention of various diseases. However, safety and efficacy of herbal medicine should be confirmed. Utilization of knowledge about positive pharmacological effects of edible plants included in in a pharmacopeia is one of probably safe and effective way for development of functional products with new beneficial effects (Kunakova et al., 2011).

Searching for new food supplements is essential. Systematic explorations of pharmacopoeial plants are one particularly relevant opportunity in Eastern Europe, especially in those areas which, for historical reasons, remain relatively isolated. The use and importance of Russian medicinal plants remains largely unknown in the West. The aim of this review is to summarize data about species that are referenced in the Russian Pharmacopoeia and about their health food value with a primary focus on literature published in Russian.

## Overview of species from the Russian pharmacopoeia used as foods

For centuries the multinational population of Russia has used plants in daily diet and for self-medication. The beneficial effects of plants were carefully collected by “knowledgeable experts” (znahar = “знахарь”in Russian) and recorded in chronicles and manuscripts. In 1778, Russia was among the first countries to implement a national pharmacopeia: The Pharmacopoeia Rossica in St. Petersburg. This work contains 770 monographs, of which 316 texts are on herbal medicinal preparations (Shikov et al., 2014a).

Since 1990, the Russian Federation has followed the State Pharmacopoeia of the USSR (1990) 11th edition. This Pharmacopeia includes 83 individual monographs for plants describing 119 species. There are 26 Rosaceae, 12 Compositae (syn. Asteraceae), eight Lamiaceae, six Polygonaceae, four each of the Apiaceae, Betulaceae, Ericaceae, and Malvaceae species, three representatives each of the Solanaceae, Leguminosae (Fabaceae), and Plantaginaceae, two species each of the Adoxaceae (Caprifoliaceae), Asparagaceae (Liliaceae, s.l), Araliaceae Fagaceae, Gentianaceae, Hypericaceae, Laminariaceae, Pinaceae, Rhamnaceae, and Violaceae, and one member each of the Acoraceae (Araceae), Brassicaceae, Caprifoliaceae (Valerianaceae), Crassulaceae, Cucurbitaceae, Cupressaceae, Equisetaceae, Hymenochaetaceae, Linaceae, Menyanthaceae, Myrtaceae, Papaveraceae, poaceae, Polemoniaceae, Ranunculaceae, Rubiaceae, Saxifragaceae, Schisandraceae, and Urticaceae, respectively.

In the most of monographs, just one species is described as a source of plant material. However, 13 species are approved for Fructus rosae, 11 species are approved for Fructus crataegi, two species are approved for Herba hyperici, Fructus alni, Herba violae etc. Different parts are described for Viburnum opulus L. (Cortex viburni as diuretic and Fructus viburni as diaphoretic and anti-inflammatory) and Crataegus (Flos crataegi−13 species and Fructus crataegi−11 species for cardiovascular problems).

The large majority of species included in the Russian Pharmacopoeia (98 out of 119) are known to have been used as food by local population in Russia. For a majority of species, the part of plant used in food and as a medicine is the same. For example, the aerial parts of *Origanum vulgare* L., *Thymus serpyllum* L., *Leonurus cardiaca* L., are used as a medicine and as a food. Acorns and leaves of *Quercus robur* L. and Q. petraea (Matt.) Liebl. are used in food, while oak bark is used as a medicine. Fruits and bark of *Viburnum opulus* L. are used as medicine, with only the fruits being edible. Roots and rhizomes of *Acorus calamus* L., *Taraxacum campylodes* G.E.Haglund (syn. *Taraxacum officinale* L.), and *Aralia elata* (Miq.) Seem. are used as a medicine while young sprouts, leaves, flowers and fruits are used in food. The aerial parts of *Equisetum arvense* L. are used in medicine, while its cone-bearing shoots and black nodules attached to the roots are eaten raw in spring.

In general, considering all 98 edible plant species in the Russian Pharmacopoeia 11th edition, a higher number of plant parts are used in nutrition as compared to medicine. In the context of this article, the term “wild medicinal plants” referrings mainly to non-cultivated species. Twenty one plants including *Calendula officinalis* L., *Eucalyptus viminalis* Labill., *Pimpinella anisum* L., *Zea mays* L., and some others do not occur in Russia outside of cultivation and are excluded from this review. All poisonous and toxic species are not used in food as such as the species belonging to the Solanaceae (*Atropa belladonna* L., *Datura stramonium* L., *Hyoscyamus niger* L.), Asparagaceae (*Convallaria majalis* L., *Convallaria keiskei* Miq.), Rhamnaceae (*Frangula alnus* Mill., *Rhamnus cathartica* L.), Ranunculaceae (*Adonis vernalis* L.), Papaveraceae (*Chelidonium majus* L.) and some species from the newly circumscribed Plantaginaceae (*Digitalis purpurea* L., *Digitalis grandiflora* Mill.).

Consumer and marketing studies invariably showed that taste, as opposed to perceived nutrition or health value is the key influence on food selection (Drewnowski and Gomez-Carneros, 2000). Sensitized to the astringent taste of tannins or some bitter poisons, humans reject foods that are perceived to be excessively astringent or bitter. *Polemonium caeruleum* L. (Polemoniaceae), *Rubia tinctorum* L. (Rubiaceae), *Alnus incana* (L.) Moench and Alnus glutinosa (L.) Gaertn. (Betulaceae) are not considered to be edible due to their taste, as well as *Gnaphalium uliginosum* L. and *Bidens tripartita* L. (Asteraceae) which, however, are without any strong taste.

Edible wild species included in the Russian Pharmacopoeia have a wide range of uses: expectorant, diuretic, astringent, haemostatic, bitterness and choleretic, anti-inflammatory, diaphoretic, tonic, sedative, spasmolytic, polyvitamin, cardiovascular, and antihelminthic (Table 1).

**Table 1:** Wild medicinal plants and their applications in food preparations.

**Species, family**	**Pharmacological group according Pharmacopoeia**	**Part described in Pharmacopoeia**	Form and dose as medicine^**^	**Part used in food**	**Application in food**	**References**
*Achillea millefolium* L., Asteraceae	Haemostatic^*^, anti-inflammatory^*^	Aerial part	Infusion (15 g in 200 ml), 65–100 ml, 2–3 times a day	Aerial part, leaves, flowers	Fresh herb added to meat and fish dishes, used as a seasoning in salads and vinaigrettes. Decoctions are added to the pastry and compotes. Dry aerial part with flowers used for liqueurs, table wines, kvass, cheese, jellies and mousses, as a spice in cooking. As a surrogate for tea. Inflorescence added to soups.	Gubanov et al., 1976; Koscheev, 1981; Grisjuk et al., 1989; Sokolov, 1993; Pieroni, 2000; Budantsev and Lesiovskaya, 2001; Soenov, 2002; Khan and Abourashed, 2010; Moerman, 2010; Kalle and Sõukand, 2012; Svanberg, 2012
*Acorus calamus* L., Acoraceae	Bitterness (appetite stimulant) and choleretic	Rhizome	Infusion (10 g in 200 ml), 25 ml, 3–4 times a day	Rhizome, leaves	Rhizome edible fresh as snack, candied as sweetmeats. Used for jam, candied peel, in confectionery, pasteurized compotes, syrup, kvass, liquors, brandy, beer, for the aromatic vinegar, as spice, for flavoring in preserves, food concentrates, for bakery products and ice cream. Rhizomes are source of starch. Leaves are edible fresh.	Annenkov, 1878; Vul'f and Maleeva, 1969; Gubanov et al., 1976; Koscheev, 1981; Kibala, 1986; Tcherepnin, 1987; Grisjuk et al., 1989; Sokolov, 1994; Budantsev and Lesiovskaya, 2001; Soenov, 2002; Moerman, 2010; Kalle and Sõukand, 2012
*Althaea officinalis* L., *A. armeniaca* Ten., Malvaceae	Expectorant	Roots	Decoction (6 g in 200 ml), 65–100 ml 3–4 times a day	Roots	Eaten cooked or roasted as vegetable. Used as additive to bread, in manufacture of sweetmeats, in home made alcohol, in soup. Infusion is added to pie and meringues. As tea substitute.	Keller, 1941; Ipatiev, 1966; Sokolov, 1985; Budantsev and Lesiovskaya, 2001; Parada et al., 2011
*Aralia elata* (Miq.)Seem., Araliaceae	Tonic^*^	Roots	EtOH (70%) tincture, 1.5–2.0 ml, 2–3 times a day	Roots	In beer, tonic alcoholic and non-alcoholic beverages.	Budantsev and Lesiovskaya, 2001; Palagina et al., 2011; Palagina and Bogoutdinova, 2012.
				Leaves, shoots	Fried and cooked as garnish.	Izmodenov, 1989; Zhuravlev and Kolyada, 1996
*Arctostaphylos uva-ursi* (L.) Spreng., Ericaceae	Diuretic	Leaves	Infusion (10 g in 200 ml), 65–100 ml, 3–5 times a day.	Berries	Eaten fresh or cooked with fish.	Holloway and Alexander, 1990; Turner et al., 2011
			Decoction (10 g in 200 ml), 15 ml 3–5 times a day		Dry in flour for bakery.	Molchanov et al., 1989
*Artemisia absinthium* L., Asteraceae	Bitterness (appetite stimulant) and choleretic	Aerial part & leaves	EtOH (70%) tincture, 0.75–1.0 ml, 3 times a day. Infusion (10 g in 200 ml), 50 ml, 3 times a day	Aerial part & leaves, inflorescences	Spicy seasoning to food, in the alcoholic (flavoring of liqueur, absinthe, wine, grappa) and non-alcoholic beverages, substitute of hop in home brewing. Essential oil is used for flavoring of ice cream, puddings, jellies, candies, and bakery. Inflorescences are used as a substitute for tea.	Ipatiev, 1966; Vul'f and Maleeva, 1969; Gubanov et al., 1976; Koscheev, 1981; Sokolov, 1993; Pieroni, 2000; Budantsev and Lesiovskaya, 2001; Khan and Abourashed, 2010; Parada et al., 2011; Kalle and Sõukand, 2012.
*Bergenia crassifolia* (L.) Fritsch, Saxifragaceae	Astringent, for external use	Rhizome	Decoction (10 g in 200 ml), 15–30 ml 3 times a day	Rhizome Leaves	Rhizomes eaten as a substitute for bread. Dry leaves ground into flour for bakery. Black leaves as tea substitute.	Vereschagin et al., 1959; Gubanov et al., 1976; Sokolov, 1987; Tcherepnin, 1987; Bugdaeva et al., 2005; Shikov et al., 2014b.
*Betula pendula* Roth, *B. pubescens* Ehrh., Betulaceae	Diuretic	Buds	EtOH (90%) tincture, 5–15 ml.	Buds	Essential oil from buds used as a flavoring in alcoholic beverage.	Gubanov et al., 1976; Fedorov, 1984; Budantsev and Lesiovskaya, 2001.
			Decoction (10 g in 200 ml), 15 ml 3–4 times a day.	Sap	Birch sap is used as a fresh drink and for preparation of kvass.	Tikhomirov, 1942; Svanberg et al., 2012.
			Infusion (10 g in 200 ml), 65–100 ml, 2–3 times a day			
*Capsella bursa-pastoris* (L.) Medik., Brassicaceae	Haemostatic^*^	Aerial part	Infusion (10 g in 200 ml), 15 ml, 4–5 times a day	Aerial part, leaves	Eaten fresh as a vegetable in salads, added to soups, borsch, schi, seasoning for meat dishes, vegetable purees and pastes, omelets, filling for pies, as a spice.	Annenkov, 1878; Tikhomirov, 1942; Klobukova-Alisova, 1958; Ipatiev, 1966; Gubanov et al., 1976; Koscheev, 1981; Reid, 1982; Sokolov, 1985; Grisjuk et al., 1989; Budantsev and Lesiovskaya, 2001; Hu, 2005; Moerman, 2010; Parada et al., 2011; Turner et al., 2011; Kalle and Sõukand, 2012; Łuczaj et al., 2013
*Carum carvi* L., Apiaceae	Spasmolytic^*^	Fruits	Infusion (20 g in 200 ml), 65–100 ml, 2–3 times a day	Fruits, roots, shoots	Spice for pickling of cabbage and cucumbers, in the production of home made cheeses, sausages, added in bread, cakes, omelets, puddings, cookies, muffins, as stuffing for dumplings. Added to soups, sauce, tea, as substitute of flour for bread. Oil from the fruits is used for flavoring of ice cream, candy, pickles, and soft drinks. Roots eaten fresh as vegetable. Young shoots cooked in soup and schi.	Keller, 1941; Tikhomirov, 1942; Gubanov et al., 1976; Koscheev, 1981; Markova et al., 1985; Tcherepnin, 1987; Sokolov, 1988; Budantsev and Lesiovskaya, 2001; Hu, 2005; Łuczaj, 2008; Moerman, 2010; Turner et al., 2011; Kalle and Sõukand, 2012; Svanberg, 2012; Sõukand et al., 2013
*Cyanus segetum* Hill (*Centaurea cyanus* L.), Asteraceae	Diuretic	Flowers	Infusion (5 g in 200 ml), 15 ml, 3 times a day	Flowers	Eaten fresh as vegetable, for side dishes decoration, for making of jelly, added to beer, as a coloring for sugar, gelatin and confectioneries, in soft drinks and alcoholic beverages.	Gubanov et al., 1976; Łuczaj, 2008; Turner et al., 2011; Łuczaj et al., 2012
*Centaurium erythraea* Rafn., *C.pulchellum* (Sw.) Druce., Gentianaceae	Bitterness (appetite stimulant)	Aerial part	Infusion (10 g in 200 ml), 65–100 ml, 2–3 times a day	Aerial part	Spice and bitterness for vermouth, aperitifs, tonic, non-alcoholic beverages. Used as substitute of hop.	Sokolov, 1990; Khan and Abourashed, 2010
*Crataegus sanquinea* Pall., *C. dahurica* Koehne ex Schneid.,	Cardiovascular	Flowers	Infusion (5 g in 200 ml), 100 ml, 3 times a day	Flowers	Eaten fresh	Sokolov, 1987
*C. chlorocarpa* Lenn, & K.Koch, *C. monogyna* Jacq., *C. rhipidophylla* Gand. *(C. curvisepala* Lindm.), *C. laevigata* (Poir.) DC., *C. pentagyna* Waldst.& Kit ex Willd, Rosaceae	Cardiovascular	Fruits	EtOH (70%) tincture, 0.5–0.75 ml. Infusion (15 g in 200 ml), 65–100 ml, 2–3 times a day.	Fruits	Eaten fresh, used for pie filling, in jelly, comfiture. Dry ground into flour for pie filling together with sugar and honey, flour added to the pastry for baking sweet bakery products, bread and pancakes, for jam, candy, sweets, biscuits. For soft drinks: kissel, compote, jelly (cooked with milk), and for wine. Whole fruits as substitute of tea and roasted as substitute of coffee.	Klobukova-Alisova, 1958; Vereschagin et al., 1959; Gubanov et al., 1976; Koscheev, 1981; Sokolov, 1987; Tcherepnin, 1987; Grisjuk et al., 1989; Efremova, 1992; Budantsev and Lesiovskaya, 2001; Pieroni et al., 2005; Khan and Abourashed, 2010; Łuczaj et al., 2012
*Equisetum arvense* L., Equisetaceae	Diuretic	Aerial part	Decoction (20 g in 200 ml), 65–100 ml, 2–3 times a day.	Aerial part, nodules	Young shoots and aerial part are eaten raw or cooked, for filling of pie, casseroles, okroshka and sauces. Nodules eaten fresh.	Berson, 1991; Moerman, 2010; Turner et al., 2011
*Helichrysum arenarium* (L.) Moench, Asteraceae	Choleretic	Flowers	Decoction (10 g in 200 ml), 100 ml, 2–3 times a day.	Flowers	Spice, flavoring additive to tea.	Facciola, 1998
*Hypericum perforatum* L., *Hypericum maculatum* Crantz., Hypericaceae	Astringent, antiseptic	Aerial part	Decoction (10 g in 200 ml), 65 ml, 3 times a day. EtOH (40%) tincture, 1.0–1.5 ml 3–4 times a day.	Aerial part	Spice seasoning for fish dishes, and sausages. In home made liquors, in alcoholic beverage industry as flavoring for bitterness brandy “Zveroboy,” “Erofeevich.” As tea substitute.	Timoshenko, 1940; Klobukova-Alisova, 1958; Makarova et al., 1960; Gubanov et al., 1976; Grisjuk et al., 1989; Koscheev, 1981; Mashanov and Pokrovsky, 1991; Budantsev and Lesiovskaya, 2001; Parada et al., 2011
*Inula helenium* L., Asteraceae	Expectorant	Roots and rhizome	Decoction (16 g in 200 ml), 100 ml, 2–3 times a day.	Roots and rhizome	Added to puddings, candy, to make jam, for soups, compotes, jelly, boiled in sugar and as substitute of ginger. To flavor vermouth, French and Swedish absinthe. Essential oil for flavoring of alcoholic beverages (spirits, beer), soft drinks, candy, cakes, ice cream, jelly, sausage, fish sauce.	Klobukova-Alisova, 1958; Ipatiev, 1966; Vul'f and Maleeva, 1969; Gubanov et al., 1976; Koscheev, 1981; Grisjuk et al., 1989; Sokolov, 1993; Budantsev and Lesiovskaya, 2001; Khan and Abourashed, 2010; Parada et al., 2011
*Juniperus communis* L., Cupressaceae	Diuretic	Fruits	Infusion (10 g in 200 ml), 15 ml, 3–4 times a day.	Fruits	Eaten fresh, used for preparation of juniper kvass, morse, beer, gin, liquors. Added as flavor and condiment in soup, pickles, sour cabbage, mincemeat, pate, wildfowl and meat dishes. In production of molasses, honey cake, candy, as coffee substitute. Extracts and essential oil added to ice cream, sausages, meat products, jelly, candy, bakery, alcoholic and non-alcoholic beverages.	Keller, 1941; Klobukova-Alisova, 1958; Gubanov et al., 1976; Koscheev, 1981; Tcherepnin, 1987; Grisjuk et al., 1989; Budantsev, 1996; Pieroni, 2000; Budantsev and Lesiovskaya, 2001; Khan and Abourashed, 2010; Moerman, 2010; Parada et al., 2011; Turner et al., 2011; Łuczaj et al., 2012; Svanberg, 2012
*Ledum palustre* L., Ericaceae	Expectorant	Shoots	Infusion (10 g in 200 ml), 50 ml, 2–3 times a day.	Shoots Leaves	Substitute of hop for beer. Substitute of tea.	Sokolov, 1985; Budantsev and Lesiovskaya, 2001.
*Leonurus cardiaca* L., *L. quinquelobatus* Gilib., Lamiaceae	Sedative	Aerial part	Infusion (15 g in 200 ml), 100 ml, 2 times a day. EtOH (70%) tincture, 0.75–1.25 ml 3–4 times a day.	Flowers, aerial part	Fresh or dry flowers as condiment in soup with legumes especial in lentil or split peas, for flavoring of beer and tea. Aerial part is used in schi.	Annenkov, 1878.
*Matricaria chamomilla* L. (*Matricaria recutita* L.), Asteraceae	Anti-inflammatory, spasmolytic	Flowers	Decoction (10 g in 200 ml), 65–100 ml, 2–3 times a day.	Flowers	Substitute of tea, added to liquor and bitter brandy, vermouth. Essential oil for enhance of fruity flavor of ice cream, candies, bakery, chewing gum, puddings, jellies.	Vul'f and Maleeva, 1969; Rubtsov, 1971; Gubanov et al., 1976; Sokolov, 1993; Pieroni, 2000; Budantsev and Lesiovskaya, 2001; Khan and Abourashed, 2010; Parada et al., 2011; Kalle and Sõukand, 2012; Svanberg, 2012
*Menyanthes trifoliata* L., Menyanthaceae	Bitterness (appetite stimulant) and choleretic	Leaves	Infusion (10 g in 200 ml), 65–100 ml, 3 times a day.	Leaves	Substitute of hop for beer. As substitute of tea.	Kalle and Sõukand, 2012
*Origanum vulgare* L., Lamiaceae	Expectorant	Aerial part	Infusion (10 g in 200 ml), 100 ml, 2 times a day.	Aerial part	Fresh leaves and tender shoots are boiled and eaten as green vegetables; fresh and dry leaves and flowers as spice and condiment in salads, soup, stews meat, sausages, casseroles, sauces, toppings and egg dishes, pickling olives, cucumber, mushrooms, and tomatoes, bakery. In soft drinks, kvass, beer, vine, brandy and liquors. As tea substitute.	Annenkov, 1878; Klobukova-Alisova, 1958; Ipatiev, 1966; Vul'f and Maleeva, 1969; Rubtsov, 1971; Gubanov et al., 1976; Koscheev, 1981; Boeva et al., 1984; Kibala, 1986; Grisjuk et al., 1989; Sokolov, 1991; Pieroni, 2000; Budantsev and Lesiovskaya, 2001; Khan and Abourashed, 2010; Parada et al., 2011; Kalle and Sõukand, 2012; Svanberg, 2012; Sõukand et al., 2013
*Panax ginseng* C.A.Mey., Araliaceae	Tonic	Rhizome	EtOH (70%) tincture, 0.375–0.625 ml 3 times a day	Roots	Added to candy, jam, chewing gum, blended coffee, bakery, canned chicken, soups, porridge, snacks, sweets, honey, punch, soft and alcoholic beverages.	Grushvitsky, 1961; Budantsev and Lesiovskaya, 2001; Khan and Abourashed, 2010; FAO/WHO, 2012b; CODEX STAN 321-2015, 2015
*Persicaria bistorta* (L.) Samp. (*Polygonum bistorta* L.), Polygonaceae	Astringent	Rhizome	Infusion (10 g in 200 ml), 100 ml, 2–3 times a day.	Rhizome	Eaten roasted, baked, cooked, as additive to rye flour, in alcoholic beverages.	Gubanov et al., 1976; Fedorov, 1984; Tcherepnin, 1987; Moerman, 2010
*Persicaria hydropiper* (L.) Delabre. (*Polygonum hydropiper* L.), Polygonaceae	Haemostatic	Aerial part	Infusion (20 g in 200 ml), 65 ml, 3–4 times a day.	Aerial part	Spice and condiment to meat, ragout. Young shoots cooked, roasted and eaten as vegetable.	Ipatiev, 1966; Vul'f and Maleeva, 1969; Reid, 1982; Fedorov, 1984; Budantsev and Lesiovskaya, 2001; Hu, 2005; Moerman, 2010
*Persicaria maculosa* Gray. (*Polygonum persicaria* L.), Polygonaceae	Astringent, hemostatic, diuretic^*^	Aerial part	Infusion (20 g in 200 ml), 15 ml, 3 times a day.	Aerial part	Eaten fresh as snack, added to salad, soup, stews, casseroles, roasted or cooked and served with oil or vinegar; in alcoholic beverages.	Reid, 1982; Fedorov, 1984; Turner et al., 2011
*Picea abies* (L.) H.Karst., Pinaceae	Anti-inflammatory	Cones	Infusion (30 g in 200 ml), external application.	Cones	As tea surrogate.	Budantsev, 1996; Budantsev and Lesiovskaya, 2001
*Pinus sylvestris* L., Pinaceae	Expectorant	Buds	Decoction (10 g in 200 ml), 65–100 ml, 2–3 times a day.	Buds, needles, sprouts	Essential oil from needles for flavoring of soft and alcoholic beverages, ice cream, jelly, pudding. Buds for beer. Sprouts in infusion as source of vitamine C. Fresh sapwood is edible and added in flour.	Vereschagin et al., 1959; Berson, 1991; Khan and Abourashed, 2010
*Plantago major* L., Plantaginaceae	Expectorant	Leaves	Decoction (10 g in 200 ml), 65–100 ml, 3–4 times a day.	Leaves	Fresh young leaves are eaten as a green vegetable in salads, added to soup, borsch, omelets, quiches, porridge, mashed potatoes, and cutlet. Dry leaves as surrogate of tea.	Tikhomirov, 1942; Klobukova-Alisova, 1958; Efremova, 1967; Rubtsov, 1971; Gubanov et al., 1976; Reid, 1982; Tcherepnin, 1987; Grisjuk et al., 1989; Sokolov, 1990; Pieroni, 2000; Budantsev and Lesiovskaya, 2001; Hu, 2005; Moerman, 2010; Parada et al., 2011; Turner et al., 2011; Kalle and Sõukand, 2012
*Polygonum aviculare* L., Polygonaceae	Diuretic^*^	Aerial part	Infusion (15 g in 200 ml), 65-100 ml, 2-3 times a day.	Aerial part	Fresh young leaves eaten as a green vegetable in salads, added to soup, pottage, condiment for meat and fish. Leaves as substitute of tea.	Reid, 1982; Fedorov, 1984; Tcherepnin, 1987; Grisjuk et al., 1989
*Prunus padus* L. (*Padus racemosa* (Lam.) Gilib., *Padus avium* Mill., *P. asiatica* Kom.), Rosaceae	Astringent	Fruits	Infusion (10 g in 200 ml), 100 ml, once a day.	Fruits	Eaten fresh as snack; dry fruits as substitute of tea. Flour from dried fruit is used in confectionery and bakery, cheesecakes, cookies, pancakes, bread (added to the grain flour), and for pie filling. Fresh and dry fruits used in kissel, jelly, jam, compotes, liquors and brandy.	Klobukova-Alisova, 1958; Vul'f and Maleeva, 1969; Gubanov et al., 1976; Koscheev, 1981; Markova et al., 1985; Sokolov, 1987; Tcherepnin, 1987; Grisjuk et al., 1989; Toren, 1996; Budantsev and Lesiovskaya, 2001; Soenov, 2002; Łuczaj, 2008; Turner et al., 2011; Kalle and Sõukand, 2012; Svanberg, 2012
*Sedum roseum* (L.) Scop. (*Rhodiola rosea* L.), Crassulaceae	Tonic	Rhizome & roots	EtOH (70%) extract, 0.125–0.25 ml 2–3 times a day.	Rhizome & roots.	Cooked and used wit butter as side dishes to meat and fish; in tonic beverages. As additive to tea.	Sokolov, 1990; Soenov, 2002
				Leaves	Young shoots and leaves in salad.	Saratikov, 1974
*Rosa majalis* Herrm., *R. acicularis* Lindl., *R. laxa* Retz., *R. davurica* Pall., *R. rugosa* Thunb., *R. canina* L., *R. corymbifera* Borkh., *R. tomentosa* Smith., *R. micrantha* Smith., Rosaceae	Polyvitamin	Fruits	Infusion (10 g in 200 ml), 100 ml, 2–3 times a day.	Fruits	Eaten fresh as snack, for pie and dessert filling, added to salads, used in puree and syrup preparation. Used in jam, marmalade, paste, comfiture, kissel, compote, and for vinegar, wine and brandy. Dry added to soup and in flour for bakery. Whole fruits as substitute of tea and roasted as substitute of coffee.	Tikhomirov, 1942; Klobukova-Alisova, 1958; Baikov, 1968; Vul'f and Maleeva, 1969; Gubanov et al., 1976; Koscheev, 1981; Markova et al., 1985; Sokolov, 1987; Tcherepnin, 1987; Grisjuk et al., 1989; Efremova, 1992; Pieroni, 2000; Budantsev and Lesiovskaya, 2001; Soenov, 2002; Hu, 2005; Pieroni et al., 2005; Khan and Abourashed, 2010; Turner et al., 2011; Kalle and Sõukand, 2012; Sõukand et al., 2013; Novruzov, 2014
*Sambucus nigra* L., Adoxaceae	Diaphoretic (sudorific)	Flowers	Infusion (5 g in 200 ml), 65–100 ml, 2–3 times a day.	Flowers	Eaten fresh, in salads. As flavoring in jelly, jam, bread, bakery, pudding, ice cream, for soft drinks and in wine, liquors, rafatia, and brandy. Young inflorescences fried in batter eaten as a dessert or as a side dish to meat. As tea substitute. Fruits for kissel, syrup, jelly, jam, and soft drinks.	Nekrasova, 1958; Pojarkova, 1958; Vul'f and Maleeva, 1969; Koscheev, 1981; Khan and Abourashed, 2010; Vallès et al., 2010; Turner et al., 2011; Lim, 2012; Svanberg, 2012; Sõukand et al., 2013 Berson, 1991
*Schisandra chinensis* (Turcz.) Baill., Schisandraceae	Tonic	Seeds	EtOH (95%) tincture, 0.5–0.75 ml 2–3 times a day.	Fruits	Eaten fresh or dry. Dry fruits and juice for sweets, soft drinks, kissel, jams, syrups, and flavoring extracts. In wine industry; as additive to tea. Dry fruits in bakery products.	Gubanov et al., 1976; Fedorov, 1984; Budantsev and Lesiovskaya, 2001; Hu, 2005; Panossian and Wikman, 2008; Khan and Abourashed, 2010; Smertina et al., 2011
*Sorbus aucuparia* L., Rosaceae	Polyvitamin	Fruits	Decoction (10 g in 200 ml), 100 ml, 3 times a day.	Fruits	Eaten fresh. Used in confiture, paste, marmalade, jam, sweet, kissel, jelly, juice, kvass, compote, candy, for pie filling. Flour from dried fruits added in bread. Added in wine, brandy. Dry as tea substitute.	Klobukova-Alisova, 1958; Vul'f and Maleeva, 1969; Koscheev, 1981; Sokolov, 1987; Grisjuk et al., 1989; Toren, 1996; Budantsev and Lesiovskaya, 2001; Soenov, 2002; Łuczaj, 2008; FAO/WHO, 2012a; Kalle and Sõukand, 2012
*Tanacetum vulgare* L., Asteraceae	Antihelminthic and choleretic	Flowers	Infusion (10 g in 200 ml), 15 ml, 3 times a day.	Flowers	Dry flowers as a garnish, as ginger and cinnamon substitute, added to meat and fish, for meat preservation; for flavoring of beverages. Flowering parts and leaves as tea substitute, in liquor, brandy, as hop substitute in beer.	Annenkov, 1878; Nekrasova, 1958; Koscheev, 1981; Berson, 1991; Sokolov, 1993; Budantsev and Lesiovskaya, 2001; Parada et al., 2011; Kalle and Sõukand, 2012
*Taraxacum campylodes* G.E. Haglund. (*Taraxacum officinale* (L.) Weber ex F.H.Wigg.), Asteraceae	Bitterness (appetite stimulant) and choleretic	Roots	Infusion (10 g in 200 ml), 65 ml, 3–4 times a day.	Roots, leaves, buds	Eaten fresh in salads, milled root added to the flour for bread baking. Extract for flavoring of ice cream, sweet, cheese, jelly, pudding, bakery, and beverages. Roasted as substitute of coffee and tea. Fresh leaves in salads, buds for pickling.	Keller, 1941; Tikhomirov, 1942; Klobukova-Alisova, 1958; Vul'f and Maleeva, 1969; Rubtsov, 1971; Gubanov et al., 1976; Koscheev, 1981; Tcherepnin, 1987; Litvintsev and Koscheev, 1988; Berson, 1991; Sokolov, 1993; Budantsev and Lesiovskaya, 2001; Khan and Abourashed, 2010; Kalle and Sõukand, 2012
*Thymus serpyllum* L., Lamiaceae	Expectorant	Aerial part	Infusion (10 g in 200 ml), 15 ml, 2–3 times a day. Decoction (20 g in 200 ml), 15–30 ml, 3–5 times a day.	Aerial part	Fresh or dry as condiment in salads, sauce, for vegetarian, fish, and meat dishes; for flavoring of vinegar, cocktails, sausages, cheese, confectionery, in beverages. For pickling of cucumber. As tea substitute.	Klobukova-Alisova, 1958; Vul'f and Maleeva, 1969; Gubanov et al., 1976; Koscheev, 1981; Grisjuk et al., 1989; Sokolov, 1991; Budantsev and Lesiovskaya, 2001; Hu, 2005; Pieroni et al., 2005; Parada et al., 2011; Kalle and Sõukand, 2012; Svanberg, 2012
*Tilia cordata* Mill., *T. platyphyllos* Scop., Malvaceae	Diaphoretic (sudorific)	Flowers	Infusion (10 g in 200 ml), 200–400 ml, 2–3 times a day.	Flowers, young leaves, bast	Flowers, leaves and bast eaten fresh. Flowers for flavoring of wine, liquor, cognac. As tea substitute.	Klobukova-Alisova, 1958; Nekrasova, 1958; Gubanov et al., 1976; Sokolov, 1985; Tcherepnin, 1987; Grisjuk et al., 1989; Budantsev and Lesiovskaya, 2001; Parada et al., 2011; Kalle and Sõukand, 2012
*Tussilago farfara* L., Asteraceae	Expectorant	Leaves	Infusion (5 g in 200 ml), 65–100 ml, 2–3 times a day.	Leaves	Young leaves eaten fresh in salads, soup and as vegetables. Dry as tea substitute.	Soenov, 2002; Kalle and Sõukand, 2012
*Urtica dioica* L., Urticaceae	Haemostatic	Leaves	Infusion (10 g in 200 ml), 50–100 ml, 3–5 times a day. EtOH (70%) extract, 0.625–0.75 ml 3 times a day.	Leaves	Leaves eaten fresh in salads, cooked as vegetables; in soup, schi, borsch, pottage, omelet, porridge, puree, filling for tortellini, component of sauces, and condiments. Milled added in flour for bakery. For juice, syrup, balsam, cocktail. Dry as tea substitute.	Tikhomirov, 1942; Klobukova-Alisova, 1958; Nekrasova, 1958; Ipatiev, 1966; Gubanov et al., 1976; Koscheev, 1981; Fedorov, 1984; Litvintsev and Koscheev, 1988; Toren, 1996; Pieroni, 2000; Budantsev and Lesiovskaya, 2001; Khan and Abourashed, 2010; Moerman, 2010; Parada et al., 2011; Turner et al., 2011; Kalle and Sõukand, 2012; Svanberg, 2012; Łuczaj et al., 2013
*Vaccinium myrtillus* L., Ericaceae	Astringent	Fruits	Eaten fresh and dry	Fruits	Eaten fresh and dry, as side dishes. Used in jam, confiture, compote, marmalade, extracts, syrup, morse, kissel, juice, compote, wine, brandy. Milled added in flour for pancakes, pie, jelly, confectionery.	Gubanov et al., 1976; Koscheev, 1981; Sokolov, 1985; Grisjuk et al., 1989; Pieroni, 2000; Budantsev and Lesiovskaya, 2001; Łuczaj, 2008; Khan and Abourashed, 2010; Kalle and Sõukand, 2012; Łuczaj et al., 2012; Svanberg, 2012
*Vaccinium vitis-idaea* L., Ericaceae	Diuretic	Leaves	Decoction (6 g in 200 ml), 65–100 ml, 2–3 times a day.	Leaves Fruits	As tea surrogate. As tea surrogate, in juice.	Klobukova-Alisova, 1958; Efremova, 1967, 1992; Krylov, 1972; Sokolov, 1985; Grisjuk et al., 1989; Toren, 1996; Budantsev and Lesiovskaya, 2001; Stryamets et al., 2015
*Valeriana officinalis* L., Caprifiolaceae	Sedative	Rhizome & roots	Infusion (20 g in 200 ml), 30–45 ml, 3–4 times a day. EtOH (70%) tincture, 0.625–0.75 ml 3–4 times a day.	Rhizome & roots	Added to soup; for pie filling, as condiment to stew meat. Extracts and essential oil as flavoring in ice cream, sweets, bakery, jelly, pudding, soft drinks, liquor, beer.	Wustenfeld and Gesler, 1959; Mashanov and Pokrovsky, 1991; Khan and Abourashed, 2010
*Viburnum opulus* L., Adoxaceae	Hemostatic	Bark	Decoction (10 g in 200 ml), 15–30 ml, 3–4 times a day.	Fruits	Eaten fresh (after freezing). Used in porridge, bakery, confiture, jam, compotes, kissel, marmalade, paste, juice, morse, mousse, vinegar, for filling of pie and sweets. As condiment to meat. As substitute of tea, and roasted as substitute of coffee.	Baikov, 1968; Gubanov et al., 1976; Tcherepnin, 1987; Grisjuk et al., 1989; Sokolov, 1990; Toren, 1996; Budantsev and Lesiovskaya, 2001; Smertina et al., 2011
	Diaphoretic, anti-inflammatory	Fruits	Infusion (10 g in 200 ml), 65 ml, 3–4 times a day.			
*Viola tricolor* L., *V. arvensis* Murray, Violaceae	Expectorant	Aerial part	Infusion (5 g in 200 ml), 100 ml, 3–4 times a day.	Flowers, leaves	Small fragrant flowers eaten fresh in salads, for dishes decoration. Cooked leaves as vegetables.	Łuczaj et al., 2013

* *Pharmacological group according to the online State Register of Medicinal preparations by the Ministry of Public Health of the Russian Federation^1^*.

** *Formulation and dose as described in Sokolov (2000)*.

*Compote – a beverage with fresh, dried or frozen fruit (whole or cut into pieces) and/or berries that's slowly cooked in a sweet water*.

*Kvass – national beverage, from sour rye flour or from baked rye bread with malt*.

*Okroshka – cold soup with kvass, meat, green vegetables and boiled egg*.

*Schi – a soup with meat or meatless, from cabbage (chopped or sour); sometimes cabbage is replaced with sorrel*.

*Borsch – a schi with beetroot, beef or pork, or lard*.

Plants with expectorant properties alleviate the symptoms of patients in certain stages of bronchitis or tracheitis. Beneficial expectorant effects are mentioned for nine edible wild species. Certain edible species (nine species) are natural diuretics. Astringent species (eight species) provide not only specific taste for wine, tea, and other beverages due to high tannins content, but are effective in stopping the flow of blood or other secretions, and resolve diarrhea. Bitterness and choleretic herbs (seven species) are useful for development of functional foods with digestion-enhancing properties. The haemostatic effects of herbs (six species) are often due to mechanisms such as tannin astringency rather than enhancement of coagulation, although there are a few haemostatic herbs have been shown to reduce clotting times and have inhibitory effects on the Platelet Aggregation Factor. Four species included in the Russian Pharmacopoeia are used for their anti-inflammatory action. In the last decades, so-called natural anti-inflammatory diets have gained popularity (Sears, 2015). Diaphoretic herbs (four species) induce involuntary perspiration and thus reduce fever, promote circulation, relieve muscle tension, aching joints, and inflammatory skin conditions. These herbs are used as well to relieve diarrhea, dysentery, kidney nephritis, liver, urinary, and gall bladder. Tonic herbs (four species) as supplement to food are consumed throughout the world on a daily basis to promote radiant health and are included in many beverages. Motherwort and valerian provide mild sedative effects. The species with spasmolytic effects (two species) are noticeable as components of diets for treatment of gastro-intestinal ailments.

Hawthorn berries and flowers are useful for the creation of functional products with cardiovascular properties. Antihelmintic properties of tansy are complementary to its choleretic effects. The well documented anxiolytic effect of *L. cardiaca* L. may lead to the development of antistress functional food products. Increased access to information through systematic evaluation of medicinal plants and its nutritional properties will support the generation of ideas for functional food with new value added properties.

Underground parts like roots, shoots and leafy greens, berries and other fleshy fruits, seeds, and other species commonly yield wild-harvested foods, a practice which is based on specialized cultural knowledge of their harvesting, preparation, cooking and other forms of processing.

## Edible wild plants–approaches and methods for assessing uses and evidence

Based on the State Pharmacopoeia of the USSR, we selected seventy wild species that are used as food in Russia and systematically searched the scientific literature (published between 1878-2016) for data using the Russia-wide applied online database E-library.ru, library catalogs of public libraries in St-Petersburg, the databases Scopus, Web of Science, PubMed, and search engine Google Scholar. The primary search criterion was a food application of medicinal plants.

Edible wild plants include food categories familiar to everyone: green vegetables and potherbs, wild berries and fruits, beverages, tea and coffee substitutes, seasonings and spices, sweets, bread surrogates, plants used for preserves. Family names of the species are based on www.theplantlist.org database with the names in the Russian Pharmacopoeia given in brackets (Table 1). Also included are the applications in food.

### Green vegetables and potherbs

This category includes the majority of examined plants. Especially in the spring or at the beginning of their growing season, many wild species produce tender, edible leaves and shoots, and flowers at the beginning of flowering. Some, like shepherd's-purse (Capsella bursa-pastoris (L.) Medik.), cornflower (*Cyanus segetum Hill*., syn. *Centaurea cyanus* L.), and broadleaf plantain (*Plantago major* L.) can be eaten raw, after being peeled from soil (Sokolov, 1985, 1990; Tcherepnin, 1987; Grisjuk et al., 1989; Budantsev and Lesiovskaya, 2001; Turner et al., 2011), whereas others, like stinging nettles (*Urtica dioica* L.) must be steamed or cooked in some way (Litvintsev and Koscheev, 1988; Fedorov, 1984; Toren, 1996). These plants are used for salads, added to soups, borsch, omelets, cooked and used as a garnish.

Many edible greens emerging right after the melting of snow are particularly important for their vitamin C content in the spring and traditionally have been used to prevent and alleviate scurvy. In particularly, soup and borsch with nettles are popular not only among rural but also many urban people (Gubanov et al., 1976; Koscheev, 1981; Tcherepnin, 1987; Litvintsev and Koscheev, 1988).

Tonic properties of shoots of aralia (*Aralia elata* (Miq.)Seem.) were recognized by Ussuri aborigines (Far East). Young shots no more than 20 cm are cooked and served as garnish (Izmodenov, 1989).

### Wild berries and fruits

Presumably, wild berries and fruits are the most favored group of edible medicinal plants in Russia, and today probably used most frequently. The wild fruits most commonly collected in Russia include bilberry (*Vaccinium myrtillus* L.), hawthorn (*Crataegus* spp.), and rose hips (*Rosa* spp.) (Gubanov et al., 1976; Koscheev, 1981; Sokolov, 1987; Tcherepnin, 1987; Grisjuk et al., 1989; Efremova, 1992; Budantsev and Lesiovskaya, 2001; Soenov, 2002; Łuczaj et al., 2012; Sõukand et al., 2013; Novruzov, 2014). All of them are consumed fresh, but some are also preserved for the winter by making jams, marmalade or pasteurized compotes (Turova and Sapozhnikova, 1989). Furthermore, they are used to make beverages. Some berries are popular among all groups of the population (like bilberry), other species that once were used as a valuable food source in some regions of Russia have seen that food use diminished because of the bitter or astringent taste as such as guelder rose (*Viburnum opulus* L.), bird cherry (*Prunus padus* L.), and rowan (*Sorbus aucuparia* L.) (Baikov, 1968; Rubtsov, 1971; Gubanov et al., 1976; Sokolov, 1985, 1990; Tcherepnin, 1987; Grisjuk et al., 1989; Toren, 1996; Smertina et al., 2011). The berries and seeds of *Schisandra chinensis* (Turcz.) Bail. were eaten by Nanai (Goldes or Samagir) hunters from Far Eastern Russia since “it gives forces to follow a sable all the day without food” and were acclaimed as a tonic, to improve night vision and to reduce hunger, thirst and exhaustion (Panossian and Wikman, 2008).

### Beverage

Aerial parts, leaves or flowers are used mainly for making beverages, while the underground parts are used rarely. Aromatic plants belonging to the Asteraceae and Lamiaceae are used to sweeten or to flavor alcoholic and non-alcoholic beverages: yarrow (*Achillea millefolium* L.), tansy (*Tanacetum vulgare* L.), and oregano (*Origanum vulgare* L.) (Timoshenko, 1940; Wustenfeld and Gesler, 1959; Koscheev, 1981; Berson, 1991; Sokolov, 1991; Budantsev and Lesiovskaya, 2001; Soenov, 2002; Kalle and Sõukand, 2012; Sõukand et al., 2013). Juicy fruits of species belonging to the Rosaceae (rowan and bird cherry) are used often for coloring of alcoholic beverages and giving a more pronounced flavor. The fruits of these plants are used both in home made alcoholic beverages as well as in wine- and brewing industry for the production of liqueurs, bitters, wines, and beers (Wustenfeld and Gesler, 1959; Gubanov et al., 1976; Koscheev, 1981; Tcherepnin, 1987; Toren, 1996; Budantsev and Lesiovskaya, 2001). Making liqueurs out of rowan berries seems to be an old habit that has been in vogue in Russia (Timoshenko, 1940). The aroma is an important attribute of refreshing soft drinks (kvass, lemonade, and fruit morse), which are popular in the summer season.

Tonic properties of ginseng and aralia were recorded in the Far Eastern Russia by hunters and their roots were regularly added to alcoholic and nonalcoholic beverages, and balsams (Grushvitsky, 1961; Zhuravlev and Kolyada, 1996; Budantsev and Lesiovskaya, 2001; Palagina et al., 2011; Palagina and Bogoutdinova, 2012).

Birch (*Betula pendula* Roth., and *B. pubescens* Ehrh.) sap has been gathered all over Russia and was usually seen as a refreshing drink (Fedorov, 1984; Tcherepnin, 1987; Budantsev and Lesiovskaya, 2001). Today, Russia, Estonia, Latvia, Lithuania, Ukraine and Belarus are the only countries where the gathering and use of birch sap has remained an important. Large birch forests, low population density and the incorporation of sap into the former Soviet economic system facilitated this (Svanberg et al., 2012). Birch sap was utilized the diverse ways as described by the Russian ethnographer Zelenin (1927). It was drunk fresh, but also fermented by adding malt, wax, beans or rye bread.

### Tea and coffee substitutes

After water, tea is the second most-consumed beverage worldwide (Keating et al., 2015). Although the English term “tea” generally denotes an infusion made of the leaves of *Camellia sinensis* (L.) Kuntze, many herbal teas, which usually are mono- or polyherbal formulations made from (medicinal) plant(s), are available worldwide. Historically in Russia such herbal teas often made from local species were widespread. Samovar and tea-drinking are an indispensable element of Russian culture. With *C. sinensis* not being available or affordable to the vast majority of population, local surrogates/substitutes of tea have been used for centuries. The best known tea surrogates were prepared from fireweed (*Epilobium angustifolium* L.) and, in Siberia - *Bergenia* spp. (Tcherepnin, 1987; Pohlebkin, 1998; Shikov et al., 2006, 2014b; Sõukand et al., 2013). Prior to the introduction of oriental (black or green) tea in Europe, *E. angustifolium* was esteemed as the “original Russian tea” and used widely throughout Russia and beyond (Litvintsev and Koscheev, 1988; Sõukand et al., 2013). However, this botanical drug is not in the Russian Pharmacopoeia. Extracts of black and fermented leaves of *B. crassifolia* are reported as appetite and energy intake suppressants (Shikov et al., 2012). Other species that are used as substitutes for tea in Russia belong to different families. First of all they are expected to bring delicious flavor, taste and nice color to the tea. Marsh Labrador tea (*Ledum palustre* L.), thyme (*Thymus serpyllum* L.), wormwood (*Artemisia absinthium* L.), are highly aromatic. Aerial parts of Saint John's wort (*Hypericum perforatum* L.), oregano, thyme, leaves of lingonberry (*Vaccinium vitis-idaea* L.), flowers of linden (*Tilia cordata* Mill. and T. platyphyllos Scop.) are among the most popular components of herbal teas in Russia (Krylov, 1972; Koscheev, 1981; Sokolov, 1985; Grisjuk et al., 1989; Efremova, 1992; Kalle and Sõukand, 2012). Dry fruits of rose hips, hawthorn, and rowan are used as substitutes of tea (Gubanov et al., 1976; Tcherepnin, 1987; Budantsev and Lesiovskaya, 2001). Ethnobotanical data also lists dried and roasted rhizomes of dandelion (*Taraxacum campylodes* G.E. Haglund), dry seeds of guelder rose, roasted fruits of hawthorn, and fruits of *R. canina* L.as coffee substitutes (Klobukova-Alisova, 1958; Vul'f and Maleeva, 1969; Koscheev, 1981; Sokolov, 1990, 1993).

### Seasoning, spices

Aromatic herbs are very important part of local gastronomy, especial for seasoning and as spices in southern Russia. In particular, sweet flag (A. calamus L.), valerian (*Valeriana officinalis* L.), elecampane (*Inula helenium* L.) roots and rhizomes, juniper (*Juniperus communis* L.), and caraway (*Carum carvi* L.) fruits, dwarf everlast (*Helichrysum arenarium* (L.) Moench) and tansy flowers, yarrow, oregano, thyme, and artemisia aerial parts are normally used as aroma and flavor enhancer or digestives (Sokolov, 1988, 1994; Berson, 1991; Mashanov and Pokrovsky, 1991). These species are used both in culinary preparations at home: they are added to the soups and main dishes, salads, meats, bakery, as well as in the food industry in the production of sausages, confectionery and bakery products.

### Sweets

The Russian make a kind of sweet flag and elecampane candied peel by boiling the transverse root slices in syrup, and drying (Gubanov et al., 1976; Koscheev, 1981; Berson, 1991). Candied fruits of hawthorn, bird cherry, guelder rose are used as sweetness (Gubanov et al., 1976; Koscheev, 1981; Tcherepnin, 1987; Budantsev and Lesiovskaya, 2001).

### Bread surrogates

In the eighteenth and the first half of the nineteenth century flour and products like bread derived from it were the main foods for Russian peasants. Consequently, every an extended period of bad weather resulting in crop failure resulted in large-scale famines. This explains why a vast variety of plant parts was used for filling bread, including roots, fruits, leaves, and barks. Roots and rhizomes of some plants are known to be good sources of starch and carbohydrates. Powdered roots of *Persicaria bistorta* (L.) Samp. were added to the rye flour for baking bread (Rubtsov, 1971; Fedorov, 1984; Tcherepnin, 1987). Bergenia rhizomes were eaten as a substitute for bread (Vereschagin et al., 1959; Tcherepnin, 1987). Flour from black leaves of bergenia is used for cookies (Bugdaeva et al., 2005). Roots of sweet flag were used as source of starch (Berson, 1991). Whole or crushed caraway seeds, dried and ground into flour fruits of hawthorn, bird cherry, rowan, guelder rose were added to the pastry when baking bread and sweet bakery (cakes and pancakes) (Gubanov et al., 1976; Koscheev, 1981; Tcherepnin, 1987; Budantsev and Lesiovskaya, 2001).

In the middle of nineteenth century, fruits of guelder rose (*Viburnum opulus*) were popular in Tver province of Russia as ingredients for making “kulaga,” a porridge based on malt flour. Guelder rose fruits tenderized with flour and honey were “a tasty cuisine and refined taste for urban residents, not excluding the nobility” (Toren, 1996). Residents of Pskov province received the nickname “kalinniki” because they cooked a delicious porridge with guelder rose (“kalina” in Russian) berries and licorice flour, which was sold at the bazaar (Toren, 1996).

Despite the fact that today wild bread additives have almost completely disappeared from the European diet (Łuczaj et al., 2012), in Russia some local bakeries produce bread and buns with nettle, hawthorn, and dog rose.

### Species used in preserves

Home made preserves have a long tradition in Russia because of the long and severe cold season. There are many canning recipes in every household for foods which have been handed down from generation to generation. Plants used for preserves can be divided into two large groups. Fresh juicy berries of rose hips, guelder rose, rowan, bird cherry, and bilberry are widely used in jam, comfiture, and marmalade. All these berries are useful for filling pies (Klobukova-Alisova, 1958; Baikov, 1968; Gubanov et al., 1976; Sokolov, 1985, 1990; Budantsev and Lesiovskaya, 2001; Soenov, 2002). The second group includes species used as spices and condiments for pickling and salting of cabbage, cucumbers, tomatoes and other vegetables, as well as mushrooms: caraway, juniper, and thyme (Rubtsov, 1971; Gubanov et al., 1976; Koscheev, 1981; Tcherepnin, 1987; Litvintsev and Koscheev, 1988; Grisjuk et al., 1989; Sõukand et al., 2013).

## Conclusions

This review focuses on botanical drugs with a monograph in the Russian Pharmacopoeia 11th edition and their potential as untapped resources beyond their strictly medical uses. In general, the Soviet period was characterized by the country being closed not only from a political point of view, but also scientifically. Most commonly, research output was only available in Russian and not translated into English dramatically restricting its availability to the international community. Here such information published in Russian was evaluated focusing on medicinal species from the Russian (Soviet) Pharmacopoeia and used as a food. This highlights the importance of the Russian Pharmacopoeia as a source of information on plant species used traditionally at the interface of food and medicine.

Clearly our approach has the limitation of only focusing on species included in the pharmacopeia and, consequently, local or endemic species are mostly excluded. However, in the context of developing high value products with potential health benefits priority needs to be given to species which are widely available and not at risk of being overexploited if the demand increases.

The evidence for the individual species varies, and while we do not claim that their use could be, based on the current data, evidence-based, the review provides a basis for further research and development.

“Functional food” are foods that not only serve to provide nutrition but also can be a source for prevention and cure of various diseases. This review highlights the potential of wild Russian species monographed in its pharmacopeia for further developing new functional foods and—through the lens of their incorporation into the pharmacopeia—showcases the species' importance in Russia.

## Author contributions

AS, AT, OP, and VM have written the first draft of the manuscript. AS, OP, and MH revised and improved the various drafts and revisions. All authors have seen and agreed on the final version of the manuscript.

### Conflict of interest statement

The authors declare that the research was conducted in the absence of any commercial or financial relationships that could be construed as a potential conflict of interest.

## Footnotes

^1^Online State Register of Medicinal preparations by the Ministry of Public Health of the Russian Federation, http://grls.rosminzdrav.ru/GRLS.aspx (accessed Aug 2017).
